# Supplementary material for: End Sequence Analysis Toolkit (ESAT) expands the extractable information from single-cell RNA-seq data
Source: Genome Res. 2016 Oct;26(10):1397–410. doi: 10.1101/gr.207902.116 (PMC5052061; doi:10.1101/gr.207902.116)
Supplement: Supplemental Material [file supp_gr.207902.116_Supp_Materials.tar.gz › ESAT-esat_v0.1/doc/html/classbroad_1_1core_1_1datastructures_1_1_pair_3_01_t1_01_4.html]

ESAT: broad.core.datastructures.Pair< T1 > Class Reference


ESAT


- Main Page
- Related Pages
- Packages
- Classes
- Files

- Class List
- Class Index
- Class Hierarchy
- Class Members

All Classes Namespaces Files Functions Variables Enumerator Pages

- broad
- core
- datastructures
- Pair< T1 >

Public Member Functions |
List of all members

broad.core.datastructures.Pair< T1 > Class Reference

|  |  |
| --- | --- |
| Public Member Functions | |
|  | Pair (T1 v1, T1 v2) |
|  | |
|  | Pair () |
|  | |
| void | setValue1 (T1 v1) |
|  | |
| void | setValue2 (T1 v2) |
|  | |
| T1 | getValue1 () |
|  | |
| T1 | getValue2 () |
|  | |
| boolean | hasValue2 () |
|  | |
| boolean | hasValue1 () |
|  | |
| boolean | isEmpty () |
|  | |
| boolean | equals (Pair< T1 > other) |
|  | |
| boolean | equals (Object other) |
|  | |
| int | hashCode () |
|  | |

## Constructor & Destructor Documentation

|  |  |  |  |
| --- | --- | --- | --- |
| broad.core.datastructures.Pair< T1 >.Pair | ( | T1 | *v1*, |
|  |  | T1 | *v2* |
|  | ) |  |  |

|  |  |  |  |  |
| --- | --- | --- | --- | --- |
| broad.core.datastructures.Pair< T1 >.Pair | ( |  | ) |  |

## Member Function Documentation

|  |  |  |  |  |  |
| --- | --- | --- | --- | --- | --- |
| boolean broad.core.datastructures.Pair< T1 >.equals | ( | Pair< T1 > | *other* | ) |  |

|  |  |  |  |  |  |
| --- | --- | --- | --- | --- | --- |
| boolean broad.core.datastructures.Pair< T1 >.equals | ( | Object | *other* | ) |  |

|  |  |  |  |  |
| --- | --- | --- | --- | --- |
| T1 broad.core.datastructures.Pair< T1 >.getValue1 | ( |  | ) |  |

|  |  |  |  |  |
| --- | --- | --- | --- | --- |
| T1 broad.core.datastructures.Pair< T1 >.getValue2 | ( |  | ) |  |

|  |  |  |  |  |
| --- | --- | --- | --- | --- |
| int broad.core.datastructures.Pair< T1 >.hashCode | ( |  | ) |  |

|  |  |  |  |  |
| --- | --- | --- | --- | --- |
| boolean broad.core.datastructures.Pair< T1 >.hasValue1 | ( |  | ) |  |

|  |  |  |  |  |
| --- | --- | --- | --- | --- |
| boolean broad.core.datastructures.Pair< T1 >.hasValue2 | ( |  | ) |  |

|  |  |  |  |  |
| --- | --- | --- | --- | --- |
| boolean broad.core.datastructures.Pair< T1 >.isEmpty | ( |  | ) |  |

|  |  |  |  |  |  |
| --- | --- | --- | --- | --- | --- |
| void broad.core.datastructures.Pair< T1 >.setValue1 | ( | T1 | *v1* | ) |  |

|  |  |  |  |  |  |
| --- | --- | --- | --- | --- | --- |
| void broad.core.datastructures.Pair< T1 >.setValue2 | ( | T1 | *v2* | ) |  |

---

The documentation for this class was generated from the following file:

- java/broad/core/datastructures/Pair.java


---

Generated on Fri Aug 15 2014 15:10:52 for ESAT by  

 1.8.7
